# The Population Health Approach: health GIS as a bridge from theory to practice

**DOI:** 10.1186/1476-072X-4-23

**Published:** 2005-10-06

**Authors:** Deborah Kelly Barnard, Weimin Hu

**Affiliations:** 1 Population Health Surveillance Unit, Vancouver Island Health Authority, Victoria, British Columbia, Canada

**Keywords:** Community Health Planning, Needs Assessment, Public Health Informatics, Medical Geography, Geographic Information Systems

## Abstract

**Background:**

The Population Health Approach, proposed by Health Canada, is the articulation of a long advocated model of human health. This approach strives to ensure that the health system is appropriately oriented to improve health status by applying evidence based practices across the continuum from health determinants to service interventions. Although conceptually appealing, it has been difficult to implement widely in the existing program-based health care system. The Population Health Surveillance Unit (PHSU) of the Vancouver Island Health Authority (VIHA) has developed a health geographical information system (HGIS), where GIS is used as both platform for information integration and as an analytical tool supporting comprehensive data analysis. With the assistance of the HGIS, the theory of the population health approach can be transformed into a practical, stepwise process supporting health services and program planning.

**Results:**

Three important components of a health service planning and evaluation framework grounded in population health theory are described in this article. In particular, a stepwise methodological process to enable the incorporation of the principles of a population health into practical applications is presented; the technical functionality to integrate multiple sources of information, with different levels and scales is discussed; and sources of information about the health of the population at the appropriate level to populate this frame are proposed. An application of the methodology in the planning of health services for a high needs neighbourhood is presented as an illustrative example.

**Conclusion:**

The population health approach incorporates the consideration of health determinants and the context within which the health conditions arise in communities. The complexity of these relationships requires the application of innovative methodologies such as Health GIS to frame the issues practically. A population health based foundation for the planning and evaluation of health services can now move from theory to practice.

## Background

**Figure 1:**
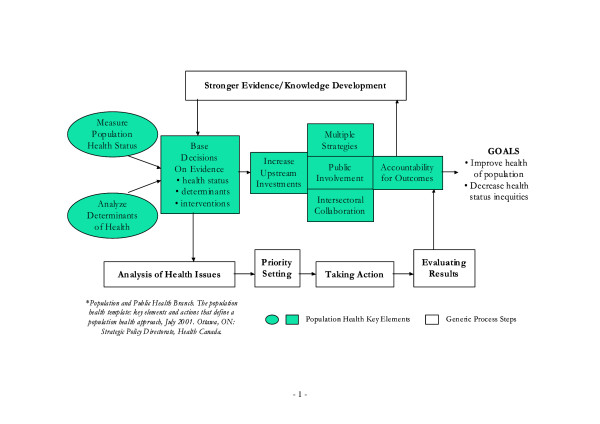
**The Population Health Approach**. The diagram depicting the Population Health Approach, proposed by the Population and Population Health Branch, Health Canada, July 2001.

The Population Health Approach, proposed by Health Canada [1], is the articulation of a long advocated model of human health [2] [Figure 1]. This approach strives to ensure that the health system is appropriately oriented to improve health status by applying evidence based practices across the continuum from health determinants to service interventions. It is a proactive stance taking responsibility for the health outcomes of a defined group of people based on a longitudinal view of the influences on health.

Although conceptually appealing, it has been difficult to implement this approach widely in the existing program-based health care system, the bridge between theory and practice tenuous [3]. In Canada, our health system has been built through the development of "sectors", a mix of publicly and privately funded services. Hospital care, physician services, laboratories and diagnostic imaging services, public health, mental health care, home and continuing care, pharmaceutical programs, workers compensation services, ambulance services, were developed as independently planned and delivered services, often with central program and policy control. It has been noted that the alignment of resources is based on factors other than overall health benefit, resulting in gaps as well as overlap and duplication. In an attempt to streamline the delivery system, making it less fragmented and more responsive to local needs, most provinces have partially devolved health delivery responsibility (for some of the publicly funded services) to regional bodies. Stated additional goals were to increase community-based services, improve public participation in health care and to encourage policies and programs to promote health [4]. Notable exclusions from the regionalized models are physician services and pharmaceutical insurance. Many of these arrangements have been in place for over a decade, yet the tension remains, with uncertainty as to the appropriate distribution of services among preventive, community- based and acute care services.

In British Columbia, despite this investment in regionalized structures, the delivery of health care information continues to be entirely program utilization based. Information development has not moved with the conceptual and structural changes in the system. This mismatch between the format and availability of information, and the tasks faced by the health authorities in the delivery of their services, is an additional impediment to the realization of integrated and efficient health services. Providing, with consistent and readily accessible information, a conceptually coherent picture of the population in terms of influences on health, health status, and the distribution and outcome of services would enable the health system to plan, implement and evaluate its services in this context.

The Population Health Surveillance Unit (PHSU) of the Vancouver Island Health Authority (VIHA) has developed a health geographical information system (HGIS), where GIS is used as both platform for information integration and as an analytical tool supporting detailed data analysis. Description of HGIS is beyond the scope of this paper and can be found in recent comprehensive review [5].

Turning data assets (large program based data sets) into person and population-based perspectives to better serve our program needs is a significant challenge. Traditional epidemiological constructs (disease rates and distributions) and utilization-based reporting (numbers and costs of services) alone cannot support the integrated view that is required for a population health approach.

With the assistance of the HGIS and the use of a wide range of data (including underutilized administrative data), the theory of the population health approach can be transformed into a practical, stepwise process supporting health services and program planning. Compelling representations can be developed of the health needs of people in the context of health influences, services and outcomes. This article describes methodology, data sources and tools, and discusses the some specific initial results obtained through the application of this process in support of the planning of services for an urban neighbourhood.

## Data and Methods

The methodology proposed for the application of population health theory involves five basic steps:

1. Population Identification

2. Population health assessment including the ecological profile

3. Description of existing service utilization and distribution

4. Analysis of the interface between needs and existing services (gaps and redundancies)

5. Outcome evaluation

These steps are iterative and cyclical, with outcomes evaluated in the context of population need. As population characteristics and service profiles shift, and more information becomes available, the framework is retained as an ongoing resource for decision-making.

### Identification of the population to be served

The Health Geographical Information System (HGIS), developed by the Population health Surveillance Unit (PHSU), uses ArcGIS spatial technology to integrate most commonly available spatial information, such as Statistics Canada's census geographies, postal codes, the boundaries of BC Ministry of Health local health authorities, and commercial spatial data including street network files. These geographies are appropriately spatially associated with each other using ArcGIS. Non-spatial attribute data, which are specific to each layer of these spatial features, are also integrated by ArcGIS and linked to specific geography. Each spatial feature and its attribute information are linked in the ArcGIS, and spatially referred to other geographical features. This HGIS plays a critical role in preliminary stages of this population health approach process.

To identify the population to be served, the HGIS is used to define the geographical boundary of the catchment area, based on pre-determined street names proposed by the project team. By overlaying other layers spatial information, such as 6-digit postal codes and census tract polygons, on the tope of the catchment area polygon, the postal codes falling within the catchment area and the census tracts are extracted and spatially referred.

BC Medical Services Plan (MSP) maintains a client registry dataset, which contains every client and related demographic and geographical information in British Columbia, who register to the MSP. The postal code of each client residential address is matched to those extracted postal codes as described previously. The clients with the postal codes of their home address matched to previously queried postal codes are then aggregated to form the population to be served. It is important to note that although the data used is at an individual level, no identifiable information is available. Additionally, the information products resulting from this work are at aggregated levels to mask any possibly identifying characteristics.

Once the base catchment population has been defined geographically, further stratification based on characteristics such as health risk factors, high co-morbidity or specific health conditions or service utilization, can be undertaken to refine health service planning.

### Population profile

The demographic characteristics of identified population are described based on the information contained in a reference client registry file. Also included in administrative data sets are the encounter records describing the interactions of individuals with physicians, hospitals and other providers. It is a significant challenge to utilize the diagnostic information contained in these administrative systems, to meaningfully describe health status. The Adjusted Clinical Group (ACG) system [6] developed by the Johns Hopkins University is used in the British Columbia Ministry of Health Services to provide a conceptually simple, statistically valid, and clinically relevant measure of population health status [7]. Measures of burden of illness and prevalence of co-morbidity of the population are also derived using the ACG system.

### Ecological setting

The ecological setting of a population describes the social and physical environmental features of the population. Routinely collected census data [8] can be used to inform these broader perspectives. The advantage of the GIS frame is that once established, other ecological features pertinent to our clearly defined cohort can be examined. The "layering" of information in this way enables a wider context than previously feasible.

As illustrative examples, socio-economic characteristics in the ecological setting are important health determinants and thus determinants of demand for health services. The national census process collects a wide array of information that is made available for analysis at aggregated levels (in this case census tracts). As described, those postal codes falling within the catchment area are spatially referenced to the census tracts within the same catchment area. The census variables associated with these census tracts, including education, income, family structure, and population living alone, are derived to describe the socio-economic characteristics of the census tracts associated with these clients residing in these areas. The choice and configuration of variables for consideration can be customized depending on their importance in the population of interest.

### Service distribution and outcomes

The existing use of services to the population of concern, are also specifically described. In British Columbia there are administrative databases that collect information about the wide array of publicly funded health services. The use of these services across the continuum of care, by the specific population of interest, provides an important view of the resources allocated. This information can now be framed with health determinants, risks and health status as well as place. Programs such as clinical services map their services to the community and understand the distribution relative both to some measures of need and other services. As evaluation is undertaken it is now appropriately anchored in an understanding of the population and outcomes can be examined through the development of more detailed models developed in this context.

## Results

The following are some sample results from work that was done to support health and social service planning for the residents of the downtown core of the downtown City A, British Columbia, Canada. Routinely available health information is reported at a "Local Health Area" level that would indicate that this area is part of a region characterized by a relatively affluent, older population with good health status. The reported experience of the care providers in this neighbourhood differs greatly from the "average" presented in these routinely available health statistics. Their anecdotal accounts describe people of all ages with complex problems and poor social circumstances and health status. Our team was asked to help delineate and quantify the needs of the population of this neighbourhood to enable specific service planning and evaluation. The specific information presented to the care providers was used as a component of a needs assessment that continues to inform planning for a continuum of health and social services in this downtown neighbourhood. This information base has supported decisions such as the location of services, composition and numbers of case management teams and spectrum of services required.

### Population identification

**Figure 5:**
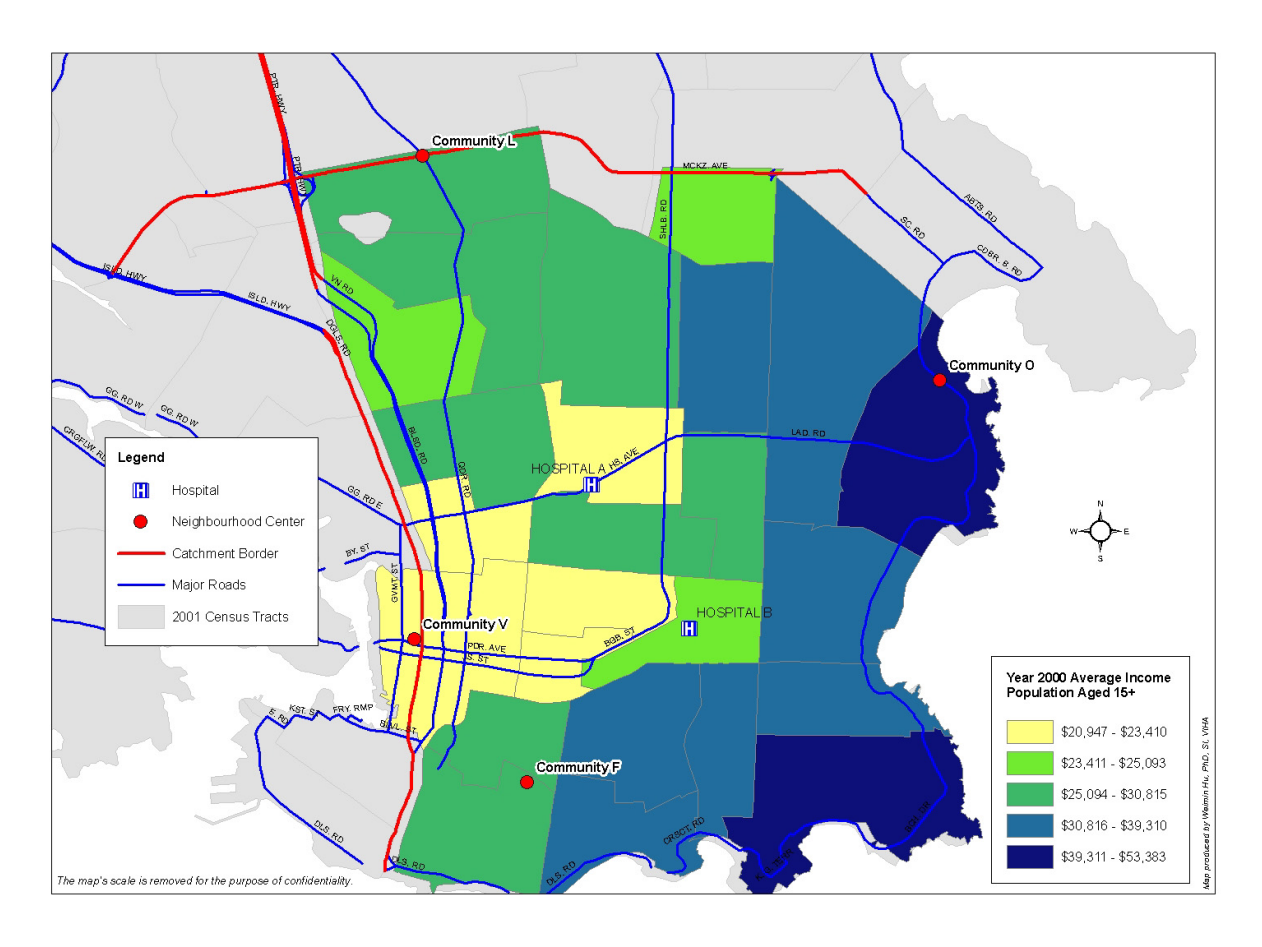
**Average Income for Population Aged 15+ by 2001 Census Tracts, City A, British Columbia, Canada**. The average income for population aged 15+ in year 2000 is displayed in different color by census tract in City A, British Columbia, Canada. The center of the City A, labeled by Community V, presenting the lowest income level, is defined as the catchment area of this study.

Figure 5 displays the geography of City A. Two layers of spatial information, average income in year 2000 for population aged 15+ by the census tracts and major streets, are overlaid. This thematic map shows a clear pattern of the distribution of annual income by the census tract and indicates the core area of downtown of the City A with the lowest average income for individuals aged 15 years over. This area fits with the general catchment described by the care providers and is thus chosen as the catchment area of this project.

**Figure 6:**
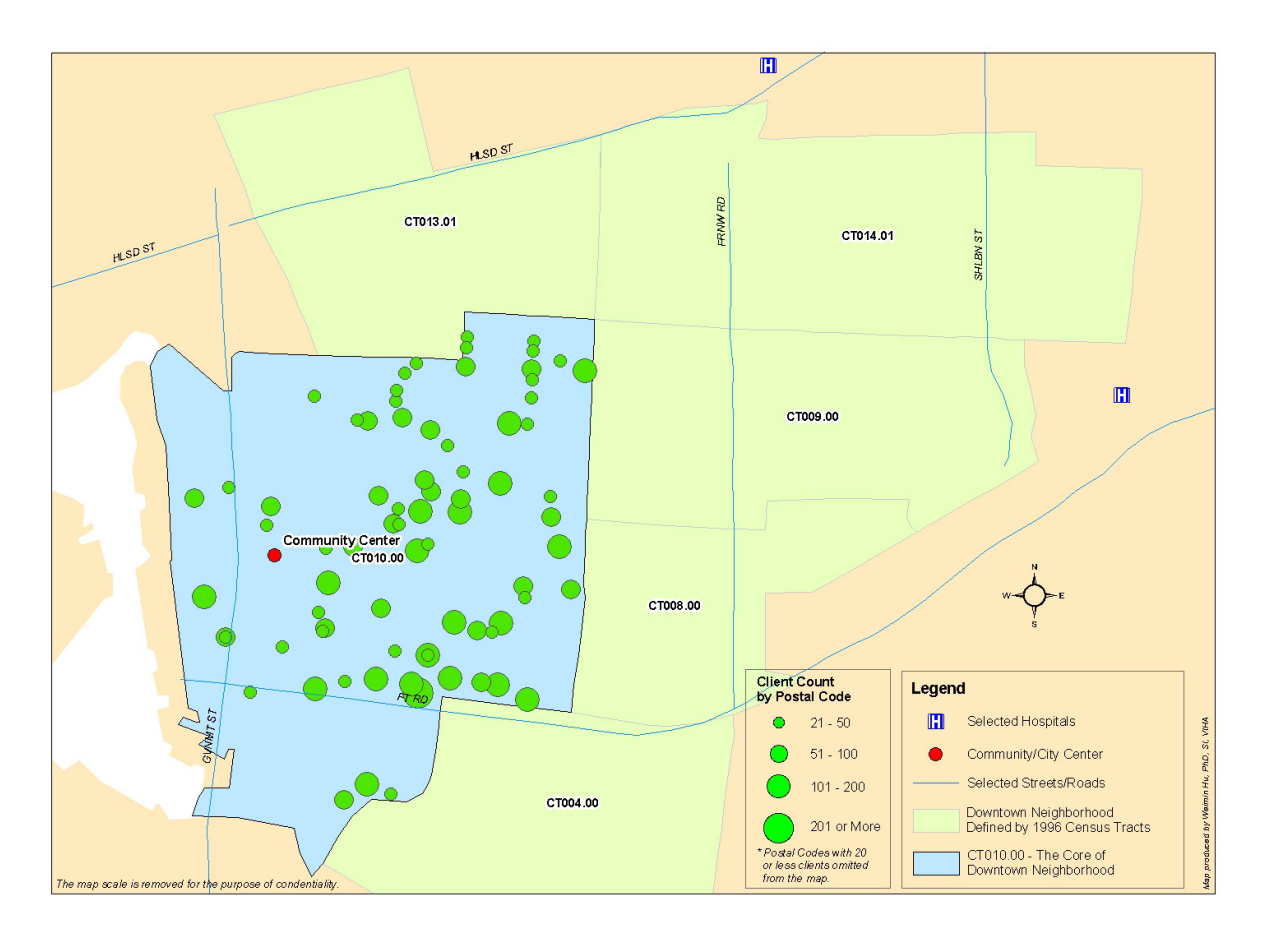
**Distribution of Clients by Postal Codes within the Core of Downtown City A (Total Clients = 6,479)**. The postal codes located within the Census Tract (CT 010.00), where the center of City A is located, are spatially queried and extracted. The queried postal codes are then matched to the residential postal codes of clients who reside in the same Census Tract. As a result, a total 6,479 clients are identified within the core of Downtown City A, and will be the population of this study. Demographic information and spatial distribution of these individual clients can be further analyzed.

Figure 6 displays the distribution of clients by their home address postal codes within the core area of Downtown City A (Census tract CT010.00), as described previously. There are 6,479 clients identified through this method.

### Population profile

**Figure 2:**
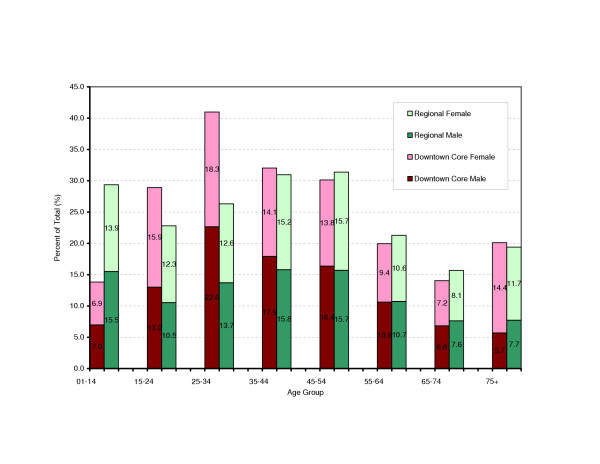
**Downtown Core Area of City A versus the Regional Total: Comparison of Clients by Age and Gender, Year 2002**. The HGIS, as a platform, integrates information on Census income, postal codes, and clients in Downtown core area of City A. The spatial analytical functions of the HGIS are used to identify and extract actual clients resided in the area (as displayed in Figure 6). These clients are then compared to total population of the region, to identify distinguishing demographic characteristics of the study population.

The demographic characteristics of this population are examined in comparison to the larger region (the entire region where City A is located). As shown in Figure 2, there is a clear difference in age distribution. Compared to the region as a whole, the service population has significant higher population at age 25–34 years (20.6% vs. 13.1%), and higher population at age groups of 25–34 and 35–44. Further analysis shows different distribution of population by gender. Males dominate in age groups 25–64 years, while there are more females in age groups 01–24 and 65–75+. For the senior population (aged 75+), females nearly triple males.

The demographic features of the population, described above, signal that this population will have an entirely different set of health needs, and thus different requirements for service delivery, compared with the larger region described by the routine administrative boundary.

**Figure 3:**
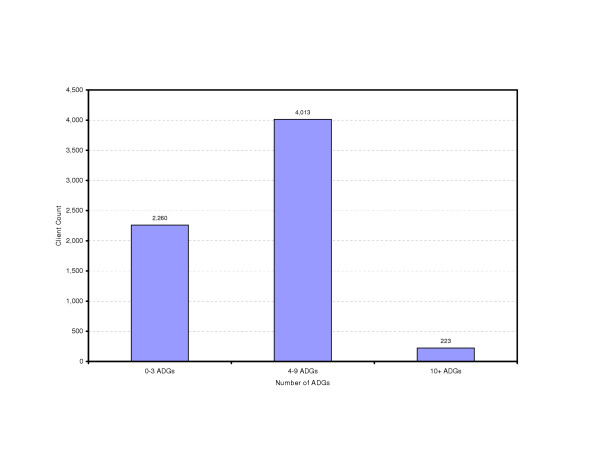
**Percent of Clients by Number of ADGs, Downtown Core Area, fiscal year 2001/2002**. The study population, identified and extracted through HGIS, is further analyzed through the Johns Hopkins ACG system. The number of ADGs (Adjusted Diagnostic Group) per capita is a method of describing the co-morbidity of a population. This figure indicates that 223 clients out of the study population (6,479 in total) have 10 or more reported health conditions, while majority of them (4,013) possesses 4–9 co-morbidities.

With the cohort defined, the postal codes can be used to link to administrative health databases to develop a profile of health characteristics. As well as examining the prevalence of individual health problems, it is important to identify the number of people who have multiple problems and the need for more complex care. This population has been stratified based on the number of health conditions associated with the individuals. This is estimated based on the number of Adjusted Diagnosis Groups and further categorized into three groups. These groups are then described by the associated resource use (Figure 3).

### Service utilization

**Figure 4:**
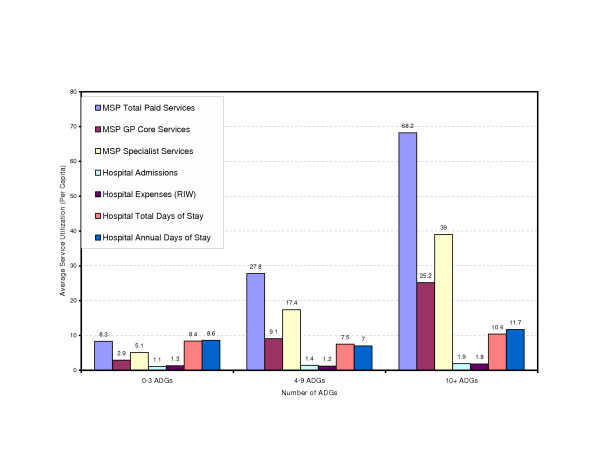
**Services Utilization by Number of ADGs for Clients of Downtown Core Area, City A, fiscal year 2001/2002**. The medical service utilization of the study population is analyzed to identify the pattern of the utilization in relation to their co-morbidities.

For this small neighbourhood there are over 200 people (Figure 3) with more than 10 diagnoses and significant hospital and physician service use (Figure 4). Interventions that are specifically disease focused will be unlikely to meet the health care needs of this group. The profile of services will also allow quantification of the resource allocation for the spectrum of health services providing a valuable support for planning and evaluation. The services supplied by the existing downtown clinic were also mapped to the population to examine issues of access.

The original plan to provide additional services based on individual program delivery in the form of a stand-alone mental health center, for example, was reevaluated based on this finding of high co-morbidity. Expanded primary health care service models [9,10] are being explored to provide more patient-based care, including case management, as a direct result of the information provided by this analytical approach.

This information now serves as a foundation for service planning based on a broad base of information about the population to be served and allows quantification of need. The service team with the responsibility for providing care for this population can continue to examine issues of pertinence and to iteratively explore information about the population, the health services and their interface. With the geographic frame it is now possible to undertake neighbourhood specific, detailed analysis of data from a variety of sources. Thus the unique functionalities of GIS enable the ongoing development and maintenance of a spatially enabled information base, which allows consideration of a broad array of data sources and pertinent information products beyond those traditionally used in health service planning. As programs are developed, the data gathered and the further analyses generated, will feed into this information repository and allow evaluations of impact and reach that are strengthened through rich context.

## Discussion and Conclusion

Three important components of a health service planning and evaluation framework grounded in population health theory have been described:

Firstly, a stepwise methodological process is delineated to enable the incorporation of the principles of a population health into practical applications.

Secondly, the technical functionality to integrate multiple sources of information, with different levels and scales is described. Geographical information systems provide this capability, building extensible profiles of populations and service delivery in context. GIS also provide unique functionality in the analysis of patterns and trends. The ability to understand groups of people through the development of information profiles unconstrained by the use of administrative boundaries and categories, transforms decision support at multiple levels of the system. GIS also can provide access tools for information products that can be useful to the full range of interested parties, from researchers and analysts to the general public.

Finally, sources of information about the health of the population at the appropriate level are identified to populate this frame. Epidemiological information from surveys and registries is not available to support population health diagnosis at the level at which service must be planned, often different from the levels delineated by existing boundaries. Through the use of the Johns Hopkins ACG system, administrative data can be sorted into meaningful information to contribute to the diagnosis of the health needs of a defined small population.

The optimal use of GIS in health applications requires the ongoing exploration of non-traditional data sources. As more administrative data becomes available, through initiatives such as the electronic health record, the utility and promise of tools such as geographical information systems will be realized. Policy and governance issues, including consideration of data access and the protection of privacy, require specific attention if these evidence-based decision support methods are to be appropriately implemented.

The population health approach incorporates the consideration of health determinants and the context within which the health conditions arise in communities. The complexity of these relationships requires the application of innovative methodologies such as Health GIS to frame the issues practically. A population health based foundation for the planning and evaluation of health services can now move from theory to practice.

## Authors' contributions

These authors contributed equally to this work.
